# Bacterial vaginosis-associated vaginal microbiota is an age-independent risk factor for *Chlamydia trachomatis*, *Mycoplasma genitalium* and *Trichomonas vaginalis* infections in low-risk women, St. Petersburg, Russia

**DOI:** 10.1007/s10096-020-03831-w

**Published:** 2020-02-08

**Authors:** Elena Shipitsyna, Tatiana Khusnutdinova, Olga Budilovskaya, Anna Krysanova, Kira Shalepo, Alevtina Savicheva, Magnus Unemo

**Affiliations:** 1 Laboratory of Microbiology, D.O. Ott Research Institute of Obstetrics, Gynecology and Reproductology, St. Petersburg, Russia; 2 Department of Clinical Laboratory Diagnostics, St. Petersburg State Pediatric Medical University, St. Petersburg, Russia; 3 WHO Collaborating Centre for Gonorrhoea and Other STIs, Department of Laboratory Medicine, Faculty of Medicine and Health, SE-701 82, Örebro University, Örebro, Sweden

**Keywords:** Bacterial vaginosis, Sexually transmitted infections, *Chlamydia trachomatis*, *Mycoplasma genitalium*, *Trichomonas vaginalis*, Low-risk women

## Abstract

The large majority of studies investigating associations between bacterial vaginosis (BV) and sexually transmitted infections (STIs) have been conducted among predominantly young women with high risk for STIs. Since a risky sexual behavior is a significant risk factor for both STIs and BV, this creates a bias toward an increased association between BV and STIs. This study evaluated associations between BV-associated vaginal microbiota and STIs (*Chlamydia trachomatis*, *Mycoplasma genitalium*, *Trichomonas vaginalis*, and *Neisseria gonorrhoeae*) in a population of women with low risk for STIs and investigated STI outcomes depending on the dominating *Lactobacillus* species. Repository cervicovaginal samples collected from reproductive-age women from January 2014 to February 2019 were characterized for vaginal microbiota types and the STIs using multiplex real-time PCR assays. In total, 95 STI-positive and 91 STI-negative samples were included. A significant, age-independent association between BV-associated vaginal microbiota and the presence of *C. trachomatis*, *M. genitalium*, and *T. vaginalis* infections was identified (age-adjusted odds ratios 2.92 [95% confidence interval (CI) 1.24–7.03], 2.88 [95% CI 1.19–7.16], and 9.75 × 10^7^ [95% CI 13.03-∞], respectively). Normal vaginal microbiota dominated by *Lactobacillus crispatus, L. gasseri,* or *L. jensenii* was a strong protective factor against *C. trachomatis* and/or *M. genitalium* infections, whereas *L. iners*-dominated microbiota was not significantly associated with *C. trachomatis* and/or *M. genitalium* positivity. The results of the present study confirm that STI prevention strategies should include interventions that also reduce the incidence of BV and promote a protective vaginal microbiota in both high- and low-risk women.

## Introduction

Sexually transmitted infections (STIs) remain a serious public health problem globally, with *Chlamydia trachomatis*, *Mycoplasma genitalium*, *Trichomonas vaginalis*, and *Neisseria gonorrhoeae* infections being the most prevalent non-viral STIs [1, 2]. In women, STIs can cause infections of lower and upper reproductive tract and result in serious complications and sequelae such as infertility, adverse pregnancy outcomes, and neonatal infections [3–6].

Bacterial vaginosis (BV) is a сommon vaginal disorder characterized by depletion of the normal *Lactobacillus* spp.-dominated microbiota and its replacement with an abundance of predominantly anaerobic commensal bacteria, of which *Gardnerella vaginalis* is considered to play a key role [7]. Clinically, BV is manifested as abnormal malodorous vaginal discharge and associated with significant reproductive morbidity [8]. The prevalence of BV in the general population is high worldwide, ranging from 23 to 29% across regions [9].

There is growing evidence that abnormal vaginal microbiota, primarily that associated with BV, might modulate susceptibility to STIs and be a risk factor for acquisition of STIs. In a recent systematic review and meta-analysis summarizing the data on association between the vaginal microbiota and STIs, a protective role of the vaginal microbiota with high *Lactobacillus* abundance was shown in relation to *C. trachomatis*, whereas no clear trend for *N. gonorrhoeae* and *M. genitalium* infections could be detected [10]. The significant association between clinical BV or BV-associated vaginal microbiota and *T. vaginalis* infection has been well documented [11–15].

The large majority of the studies that measured the association between BV (or BV-associated vaginal microbiota) and STIs have been conducted among predominantly young women with a higher risk for STIs, such as STI clinics attendees [16–18], commercial sex workers [19–21], and adolescents or young women with high-risk sexual behavior [22, 23]. Even when studies were conducted among women presenting for routine medical care [12], women recruited from primary care, gynecology, and family planning clinics [24], or rural women from community-based organizations [25], STI positivity rates ranged from relatively high to high. For example, among rural women attending mother’s clubs in Peru, who on average had one partner and therefore were considered to be of low risk in regard to STIs, the prevalence of trichomoniasis was 16.5%, *C. trachomatis* infection 6.8%, gonorrhea 1.2%, and BV (by either Nugent’s score or Amsel’s criteria) 43.7% [25]. It is well recognized that BV is associated with risky sexual behavior, and many epidemiological studies support its sexual transmission [26–28], which in populations with high risk and/or high prevalence of STIs creates a bias toward an increased association between BV and STIs.

Vaginal lactobacilli are considered to play a major role in the protection of the female reproductive tract from pathogenic microorganisms as well as BV, with the acidification of the vaginal environment being a main mechanism of the protection [29]. *Lactobacillus crispatus, L. iners*, *L. jensenii,* and *L. gasseri* are the dominating *Lactobacillus* species [30], but recent studies have suggested that their protective potentials are not equal [16, 18, 31].

The aims of the present study were to evaluate associations between BV-associated vaginal microbiota, defined using a previously validated multiplex PCR assay [32–34], and STIs (*C. trachomatis*, *M. genitalium*, *T. vaginalis,* and *N. gonorrhoeae* infections) in a population of women at low risk for STIs. In addition, we investigated STI outcomes depending on the vaginal microbiota types, with grouping the normal microbiota into four categories based on the dominating *Lactobacillus* species.

## Materials and methods

### Study design

This retrospective case-control study was performed at the D.O. Ott Research Institute of Obstetrics, Gynecology and Reproductology (the Ott Institute), St. Petersburg, Russia. Repository cervicovaginal samples from outpatients attending for routine gynecological care, which had been submitted to the Laboratory of Microbiology of the Ott Institute from January 2014 to February 2019 for diagnostic PCR testing for at least one of the non-viral STIs mentioned above, were used in the study. The routine diagnostic testing for *C. trachomatis*, *M. genitalium*, *T. vaginalis,* and *N. gonorrhoeae* had been performed using PCR assays developed by InterLabService (Moscow, Russia) and DNA Technology (Moscow, Russia), and all the used PCR assays have been previously validated in comparison with PCR assays commercially available internationally [35–38]. The cervicovaginal swabs had been collected, after removing cervical mucus, from the cervix and vagina with either two separate swabs into the same tube of transport media provided by the manufacturers of the used PCR assays (see above) or, most frequently, the same swab from both sites. The samples after routine testing had been stored at − 70 °C. The samples (STI positive cases and STI negative controls) were selected consecutively in reverse chronological order. Patients’ data (age, pregnancy status, and results of STI testing) were obtained from the database of the Laboratory of Microbiology. The inclusion criteria were reproductive age (18–50 years), not being pregnant at the time of sample collection, and not being tested positive for BV and/or any of the four STIs within 2 months prior to enrollment. Sample size was estimated with the use of Fleiss’s criterion with continuity correction at the Open Source Epidemiologic Statistics for Public Health (OpenEpi version 3.01) [39] based on assumed proportions of the main exposure (BV-associated vaginal microbiota) of 15% in controls, 40% in *C. trachomatis* and *M. genitalium* cases, and 90% in *T. vaginalis* cases. These proportions were estimated via testing the first 30 STI-negative and 30 STI-positive samples (*C. trachomatis* (*n* = 13), *M. genitalium* (*n* = 12), and *T. vaginalis* (*n* = 5)) included in the present study. None of the few *N. gonorrhoeae* samples tested positive during this period was available or eligible. The estimated minimum numbers of positive samples for overall STIs were 57, for *C. trachomatis* and *M. genitalium* 41 each*,* and for *T. vaginalis* five samples. The study was approved by the Ethical Committee at the D.O. Ott Research Institute of Obstetrics, Gynecology and Reproductology (approval number 95/2019), and waiver of informed consent was obtained.

### DNA isolation

For the present study, DNA for all PCR assays was isolated from 100 μL of sample using the silica-based manual extraction kit DNA-Sorb-AM (InterLabService, Moscow, Russia), according to the manufacturer’s instructions, with an elution volume of 100 μL. The DNA preparations were stored at 4 °C prior to amplification, which was performed within 3 days.

### PCR assay for sexually transmitted infections

Verification of the previously performed routine PCR testing results prior to including the samples in the study was performed using the internationally validated multiplex real-time PCR assay AmpliSens N.gonorrhoeae/C.trachomatis/M.genitalium/T.vaginalis-MULTIPRIME-FRT (InterLabService, Moscow, Russia) [40].

### PCR assays for characterization of the vaginal microbiota

BV-associated vaginal microbiota was detected using an internationally validated quantitative multiplex real-time PCR assay (AmpliSens Florocenosis/Bacterial vaginosis-FRT; InterLabService, Moscow, Russia), which measures the quantity of *G. vaginalis*, *Atopobium vaginae* and *Lactobacillus* spp. relative to the total bacterial load [32–34]. PCR analysis and interpretation of the results were performed according to the manufacturer’s instructions. Ratio coefficients (RC) were calculated as follows: RC1 = lg [*Lactobacillus* spp.] – lg [*G. vaginalis* + *A. vaginae*], RC2 = lg [Bacteria] – lg [*Lactobacillus* spp.], and RC3 = lg [Bacteria] – lg [*G. vaginalis* + *A. vaginae*]. A result was interpreted as normal vaginal microbiota if *G. vaginalis* and/or *A. vaginae* were absent or their cumulative concentration was less than the *Lactobacillus* spp. concentration (RC1 > 1). A sample was categorized as BV if *G. vaginalis* and/or *A. vaginae* concentrations were equal to or exceeded *Lactobacillus* spp. concentration (RC1 < 0.5). A sample was categorized as intermediate if *G. vaginalis* and/or *A. vaginae* concentrations were similar to *Lactobacillus* spp. concentration (0.5 ≤ RC1 ≤ 1). Finally, a sample was categorized as unspecified microbiota alteration if the concentration of *Lactobacillus* spp. was decreased, and the concentration of *G. vaginalis* and/or *A. vaginae* was substantially lower than the concentration of total bacteria (RC2 > 1 and RC3 > 2).

Detection and quantification of *L. iners*, *L. crispatus*, *L. jensenii*, and *L. gasseri* was performed using a research kit based on multiplex real-time PCR (DNA Technology, Moscow, Russia), which detects, except for the main four *Lactobacillus* species, also *L. vaginalis, L. acidophilus*, and *L. johnsonii*. Validation of the kit for the present study was performed using clinical isolates of numerous microbial species, including diverse *Lactobacillus* species, routinely cultured from vaginal samples and species identified using MALDI TOF mass spectrometry analysis in a Microflex instrument (Bruker Daltonics). In the studied cervicovaginal samples with normal microbiota, the dominating *Lactobacillus* species was determined.

### Statistical analyses

On the basis of PCR characterization of the vaginal microbiota, each sample was assigned to one of the following categories: BV-associated microbiota, intermediate microbiota, unspecified microbiota alteration, *L. iners-*dominated microbiota, *L. crispatus-*dominated microbiota, *L. jensenii-*dominated microbiota, and *L. gasseri*-dominated microbiota. Distributions of the microbiota types in patients with STIs (combined and individual) were compared with that in STI-negative patients using Pearson’s chi-square test. Differences in age between patients with STIs (combined and individual) and STI-negative patients were evaluated using the Mann-Whitney U test. Univariate logistic regression analysis was used to quantify the strength of association of STIs (combined and individual) with BV-associated microbiota and age and between STIs (combined) and different microbiota types. Multivariate logistic regression was used to estimate age-adjusted odds ratios. Age was included in the logistic regression models in three categories: 18–25, 26–35, and 36–50 years. Tests for significance and confidence intervals in logistic regression were likelihood ratio based. Statistical significance was defined as *p* < 0.05 (2-sided) for all analyses. Data were analyzed using JMP 14.3 (SAS Institute, Cary, NC, USA).

## Results

### Estimated STI positivity in the target population

In order to estimate the STI positivity in the target population, data extracted from the database of the Laboratory of Microbiology at the Ott Institute were analyzed. Table 1 summarizes the number and proportion of cervicovaginal samples positive for *C. trachomatis*, *M. genitalium*, *T. vaginalis*, and/or *N. gonorrhoeae* from January 2014 to February 2019. *C. trachomatis* was the most prevalent STI agent (1.4%), followed by *M. genitalium* (0.5%), *T. vaginalis* (0.3%), and *N. gonorrhoeae* (0.2%).

**Table 1:** Number and proportion of cervicovaginal samples positive for *Chlamydia trachomatis*, *Mycoplasma genitalium*, *Trichomonas vaginalis*, and/or *Neisseria gonorrhoeae* in outpatients attending for routine gynecological care from January 2014 to February 2019

Agent	No. of positive samples/No. of submitted samples (%)
*Chlamydia trachomatis*	271/19918 (1.4)
*Mycoplasma genitalium*	61/13021 (0.5)
*Trichomonas vaginalis*	12/3951 (0.3)
*Neisseria gonorrhoeae*	4/2598 (0.2)

### Selection of cases and controls

**Fig. 1:**
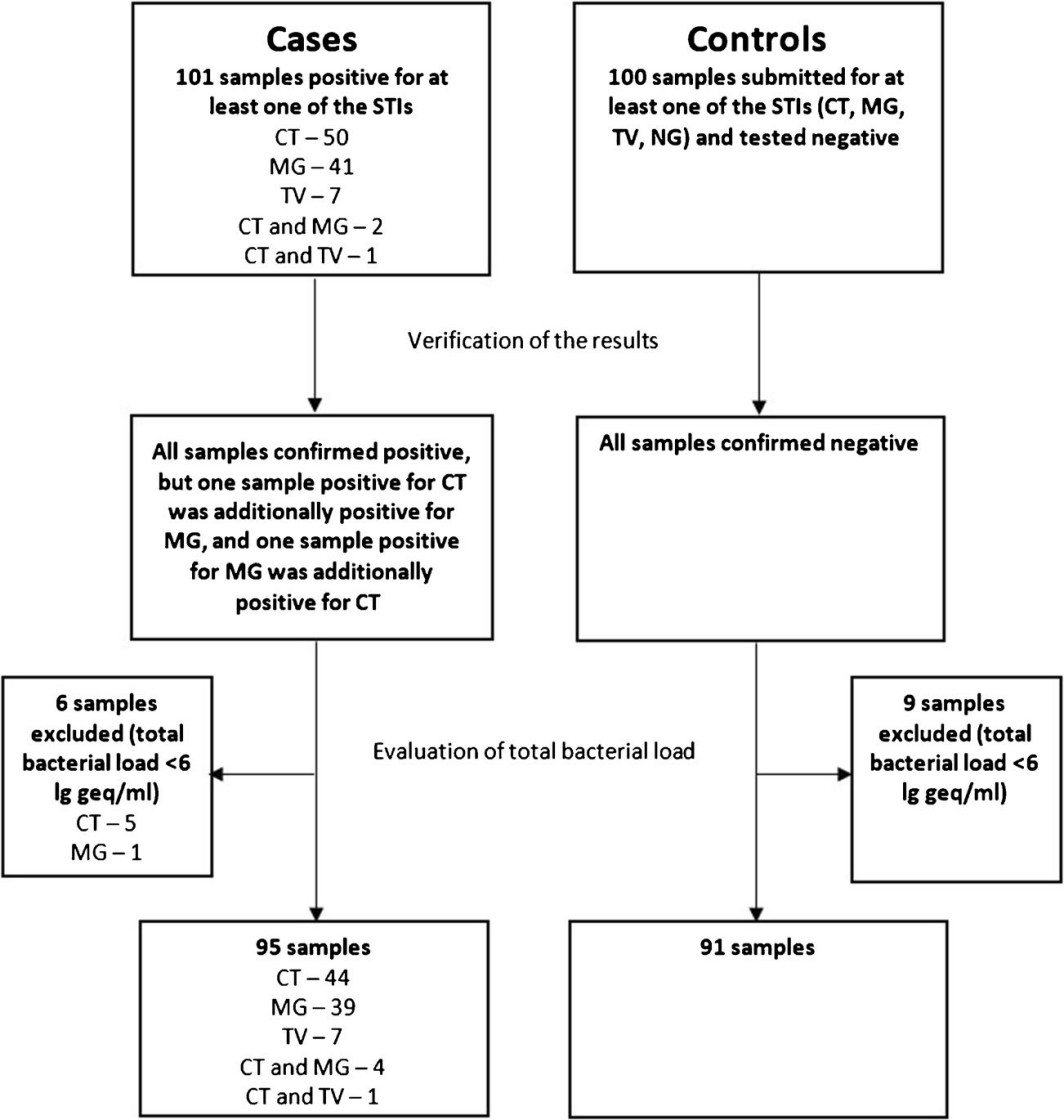
Selection of cases and controls. STI, sexually transmitted infection; CT, *Chlamydia trachomatis*; MG, *Mycoplasma genitalium*; TV, *Trichomonas vaginalis*; NG, *Neisseria gonorrhoeae*; geq, genome equivalent

A flowchart describing the selection of cases and controls for the present study is summarized in Fig. 1. In total, 101 STI-positive samples were selected. These included 50 samples positive for *C. trachomatis*, 41 samples positive for *M. genitalium*, 7 samples positive for *T. vaginalis*, 2 samples positive for *C. trachomatis* and *M. genitalium*, and one sample positive for *C. trachomatis* and *T. vaginalis*. The included *M. genitalium* and *T. vaginalis* positive samples constituted all available samples of those tested positive for these infections from January 2014 to February 2019 and meeting the eligibility criteria. The *C. trachomatis* samples represented consecutive samples selected in reverse chronological order in 2019 and 2018. None of the four *N. gonorrhoeae* positive samples were available or eligible. The control group comprised 100 consecutive samples submitted in 2019 for at least one of the STIs and tested negative.

To verify the results of previous routine diagnostic testing and to test for the STIs that were not requested, all selected samples were tested using the AmpliSens N.gonorrhoeae/C.trachomatis/M.genitalium/T.vaginalis-MULTIPRIME-FRT (InterLabService, Moscow, Russia). All cases were confirmed as positive, but one sample positive for *C. trachomatis* was additionally positive for *M. genitalium*, and one sample positive for *M. genitalium* was additionally positive for *C. trachomatis* (in both cases testing for the second infection had not been performed in the routine diagnostic testing, due to lack of request). All controls were negative for all the tested STIs.

As testing for BV-associated vaginal microbiota was PCR based, total bacterial load ≥ 6 lg genome equivalent (geq)/ml was used, as indicated in the manufacturer’s instructions, as a cut-off for sample adequacy. Consequently, 6 case and 9 control samples showing total bacterial load < 6 lg geq/ml were excluded. Thus, 95 STI-positive samples (48 samples positive for *C. trachomatis*, 43 samples positive for *M. genitalium*, and 8 samples positive for *T. vaginalis*, with 5 cases being mixed infections) and 91 STI-negative samples constituted the final cases and controls in the present study (Fig. 1).

### Validation of the multiplex real-time PCR-based research test for the differentiation and quantification of *Lactobacillus* spp.

The validation of the *Lactobacillus* research kit for the present study aimed to confirm that (1) the assay was able to reliably differentiate the four main *Lactobacillus* species and (2) it did not detect vaginal non-*Lactobacillus* species. The following clinical vaginal isolates were tested: *L. crispatus* (*n* = 9), *L. iners* (*n* = 4), *L. jensenii* (*n* = 4), *L. gasseri* (*n* = 2), *L. vaginalis* (*n* = 2), *L. johnsonii* (*n* = 2), *L. plantarum* (*n* = 2), *L. rhamnosus* (*n* = 2), *L. fermentum* (*n* = 1), *L. paracasei* (*n* = 1), *Staphylococcus aureus* (*n* = 1), *Staphylococcus haemolyticus* (*n* = 1), *Streptococcus agalactiae* (*n* = 1), *Escherichia coli* (*n* = 1), *Klebsiella pneumoniae* (*n* = 1), *Proteus mirabilis* (*n* = 1), *Enterobacter aerogenes* (*n* = 1), *Enterobacter cloacea* (*n* = 1), *Enterococcus faecalis* (*n* = 1), *Corynebacterium amycolatum* (*n* = 1), *G. vaginalis* (*n* = 1), and *Candida albicans* (*n* = 1). The test correctly detected all targeted *Lactobacillus* species. None of the non-*Lactobacillus* isolates tested positive.

### Characteristics of women with and without STIs

A total of 186 women (95 cases and 91 controls) aged 19–49 years (median age 31 years, interquartile range 27–35 years) were included. Women with STIs were overall significantly younger (median age 30 years, interquartile range 29–33 years) than women without STIs (median age 32 years, interquartile range 28–37 years; *P* = 0.0026). This difference mainly reflected the significantly younger age of women with *C. trachomatis* (median age 27 years, interquartile range 25–34 years; *P* = 0.0011) and *M. genitalium* (median age 30 years, interquartile range 26–33 years; *P* = 0.0127), whereas the age of women with *T. vaginalis* did not differ significantly from that of control women (median age 35 years, interquartile range 31–42 years; *P* = 0.1611).

Distribution of the vaginal microbiota types in women with STIs differed significantly (*P* = 0.0022) from that in women without STIs (Table 2). The most prominent differences included higher proportions of BV-associated vaginal microbiota in STI-positive women, with all 8 *T. vaginalis* samples comprising BV-associated microbiota. Furthermore, samples from STI-positive women contained much lower proportions of vaginal microbiota dominated by *L. jensenii* and *L. gasseri*. Notably, in one sample comprising normal microbiota, the only identified *Lactobacillus* species was *L. acidophilus*.

**Table 2:** Vaginal microbiota types in women with and without sexually transmitted infections (STIs)

Vaginal microbiota type	No. of samples (%)				
	STI negative (*n* = 91)	Any STI (*n* = 95)	*Chlamydia trachomatis* infection (*n* = 49)	*Mycoplasma genitalium* infection (*n* = 43)	*Trichomonas vaginalis* infection (*n* = 8)
*Normal microbiota types*					
*L. iners* dominating	27 (29.7)	34 (35.8)	17 (34.7)	19 (44.2)	0 (0)
*L. crispatus* dominating	15 (16.5)	10 (10.5)	8 (16.3)	2 (4.7)	0 (0)
*L. jensenii* dominating	13 (14.3)	3 (3.2)	1 (2.0)	2 (4.7)	0 (0)
*L. gasseri* dominating	9 (9.9)	4 (4.2)	2 (4.1)	2 (4.7)	0 (0)
*L. acidophilus* dominating	1 (1.1)	0 (0)	0 (0)	0 (0)	0 (0)
*Abnormal microbiota types*					
BV-associated microbiota	15 (16.5)	38 (40.0)	18 (36.7)	15 (34.9)	8 (100)
Intermediate microbiota	6 (6.6)	3 (3.2)	1 (2.0)	2 (4.7)	0 (0)
Unspecified microbiota alteration	5 (5.5)	3 (3.2)	2 (4.1)	1 (2.3)	0 (0)
P value*		**0.0022**	**0.0525**	**0.0487**	**0.0002**

*P* values define significance in Pearson’s chi-square tests comparing the distributions of the microbiota types in women with STIs (combined and individual) with those in STI-negative women. Significant values are in bold letters. Cases with mixed infections (*n* = 5) were included in all corresponding counts

### Association between sexually transmitted infections, age, and the vaginal microbiota

Table 3 displays the results of the univariate and multivariate logistic regression models aimed to measure the strength of association of the STIs (combined and individual) with BV-associated microbiota and age. The categories of intermediate microbiota and unspecified microbiota alteration were combined in one category, namely, abnormal non-BV microbiota. Younger age (the categories 19–25 years and, to a lesser degree, 26–35 years) was a strong predictor of both *C. trachomatis* and *M. genitalium* infections, whereas *T. vaginalis* infection was not significantly associated with age. The odds of detecting STIs, both individual and combined, was significantly higher in women with BV-associated microbiota than in women with normal microbiota (age-adjusted ORs 3.78 [95% CI 1.84–8.14], 2.92 [95% CI 1.24–7.03], 2.88 [95% CI 1.19–7.16], and 9.75 × 10^7^ [95% CI 13.03-∞] for any STI, *C. trachomatis* infection, *M. genitalium* infection, and *T. vaginalis* infection, respectively). Abnormal non-BV microbiota displayed no association with the STIs.

**Table 3:** Associations between bacterial vaginosis-associated vaginal microbiota and *Chlamydia trachomatis*, *Mycoplasma genitalium*, and *Trichomonas vaginalis* (combined and individual) in low-risk women in St. Petersburg, Russia

Characteristic	Any STI (n = 95)			*Chlamydia trachomatis* infection (n = 49)			*Mycoplasma genitalium* infection (n = 43)			*Trichomonas vaginalis* infection (n = 8)		
	No. of positive samples/No. of samples (%)	Crude OR [95% CI], *P* value	Age-adjusted OR [95% CI], *P* value	No. of positive samples/No. of samples (%)	Crude OR [95% CI], *P* value	Age-adjusted OR [95% CI], *P* value	No. of positive samples/No. of samples (%)	Crude OR [95% CI], *P* value	Age-adjusted OR [95% CI], *P* value	No. of positive samples/No. of samples (%)	Crude OR [95% CI], *P* value	Age-adjusted OR [95% CI], *P* value
*Age*												
19–25	26/38 (68.4)	**4.87 [1.90–13.24], 0.0008**		18/30 (60.0)	**8.10 [2.58–29.47], 0.0002**		10/22 (45.5)	**5.62 [1.55–24.01], 0.0080**		0/12 (0.0)	2.28 × 10^−7^ [∞-1.52], 0.0958	
26–35	57/109 (52.3)	**2.47 [1.15–5.52], 0.0193**		26/78 (33.3)	2.70 [0.99–8.68], 0.0515		29/81 (35.8)	**3.76 [1.31–13.66], 0.0121**		4/56 (7.1)	0.52 [0.11–2.35], 0.3824	
36–49 (reference)	12/39 (30.8)	1		5/32 (15.6)	1		4/31 (12.9)	1		4/31 (12.9)	1	
*Vaginal microbiota*												
BV	38/53 (71.7)	**3.23 [1.63–6.66], 0.0007**	**3.78 [1.84–8.14], 0.0002**	18/33 (54.5)	**2.79 [1.24–6.38], 0.0134**	**2.92 [1.24–7.03], 0.0140**	15/30 (50.0)	**2.60 [1.11–6.15], 0.0281**	**2.88 [1.19–7.16], 0.0194**	8/23 (34.8)	**1.17 × 10**^**8**^**[14.94-∞], < 0.0001**	**9.75 × 10**^**7**^**[13.03-∞], < 0.0001**
Abnormal non-BV	6/17 (35.3)	0.70 [0.23–1.96], 0.4963	0.82 [0.26–2.45], 0.7272	3/14 (21.4)	0.63 [0.14–2.22], 0.4936	0.80 [0.16–3.10], 0.7545	3/14 (21.4)	0.71 [0.15–2.50], 0.6111	0.87 [0.18–3.35], 0.8459	0/11 (0)	1.00 [∞-∞], 1.0000	0.85 [∞-∞], 1.0000
Normal (reference)	51/116 (44.0)	1		28/93 (30.1)	1		25/90 (27.8)	1		0/65 (0.0)	1	

*STI* sexually transmitted infection; *OR* odds ratio; *CI* confidence interval; *BV* bacterial vaginosis; Abnormal non-BV, combination of the categories for intermediate microbiota and unspecified microbiota. Significant values are in bold letters

Evaluation of STI outcomes depending on the vaginal microbiota types, with subdivision of the normal microbiota into four categories in accordance with the dominating *Lactobacillus* species, was performed in a logistic regression analysis combining *C. trachomatis* and *M. genitalium* infections (Table 4). The cases of single *T. vaginalis* infection (*n* = 7) were not included in this analysis because of comprising exclusively BV-associated microbiota. The sample with only *L. acidophilus* was not either included in this analysis. Two approaches were used, one using BV-associated microbiota as reference category and the other using *L. crispatus*-dominated microbiota as reference category. *L. crispatus, L. gasseri*, and *L. jensenii*-dominated microbiota showed strong negative association with *C. trachomatis* and/or *M. genitalium* infections when compared with BV-associated microbiota, whereas *L. iners*-dominated microbiota was not significantly associated with neither presence nor absence of the STIs. When *L. crispatus*-dominated microbiota was used as reference category, only BV-associated microbiota was significantly associated with the STIs. Although the odds of detecting STIs in *L. iners*-dominated microbiota was twice as high as that in *L. crispatus*-dominated microbiota, the difference did not reach statistical significance. Intermediate microbiota and unspecified microbiota alteration were not significantly associated with the STIs either when compared with BV-associated microbiota or *L. crispatus*-dominated microbiota.

**Table 4:** Association between vaginal microbiota types and sexually transmitted infections (*Chlamydia trachomatis* and/or *Mycoplasma genitalium*) in low-risk women in St. Petersburg, Russia

Vaginal microbiota type	No. of positive samples/No. of samples (%)	Crude OR [95% CI], *P* value	Age-adjusted OR [95% CI], *P* value
BV-associated microbiota (reference)	31/46 (67.4)	1	1
Intermediate microbiota	3/9 (33.3)	0.24 [0.05–1.10], 0.0578	0.23 [0.05–1.16], 0.0756
Unspecified microbiota alteration	3/8 (37.5)	0.29 [0.06–1.38], 0.1126	0.39 [0.08–2.00], 0.2601
*L. iners* dominating	34/61 (55.7)	0.61 [0.27–1.35], 0.2198	0.56 [0.24–1.30], 0.1777
*L. crispatus* dominating	10/25 (40.0)	**0.32 [0.12–0.89], 0.0257**	**0.27 [0.09–0.79], 0.0168**
*L. jensenii* dominating	3/16 (18.8)	**0.11 [0.03–0.45], 0.0006**	**0.12 [0.03–0.49], 0.0035**
*L. gasseri* dominating	4/13 (30.8)	**0.22 [0.06–0.81], 0.0180**	**0.20 [0.05–0.77], 0.0199**
*L. crispatus* dominating (reference)		1	1
BV-associated microbiota		**3.10 [1.13–8.51], 0.0257**	**3.65 [1.26–10.57], 0.0168**
Intermediate microbiota		0.75 [0.15–3.72], 0.7226	0.84 [0.15–4.58], 0.8399
Unspecified microbiota alteration		0.90 [0.17–4.64], 0.8996	1.44 [0.26–8.07], 0.6816
*L. iners* dominating		1.89 [0.73–4.87], 0.1840	2.06 [0.77–5.52], 0.1525
*L. jensenii* dominating		0.35 [0.08–1.53], 0.1447	0.43 [0.09–1.99], 0.2801
*L. gasseri* dominating		0.67 [0.16–2.77]. 0.5731	0.71 [0.16–3.11], 0.6542

*STI* sexually transmitted infection; *OR* odds ratio; *CI* confidence interval; *BV* bacterial vaginosis. Significant values are in bold letters

## Discussion

This study shows that BV-associated vaginal microbiota is an age-independent risk factor for *C. trachomatis*, *M. genitalium*, and *T. vaginalis* infections in women with low risk for STIs in St. Petersburg, Russia, with the strongest association demonstrated for *T. vaginalis* infection. The normal vaginal microbiota with domination of *L. crispatus*, *L. jensenii*, or *L. gasseri* had a strong negative association with *C. trachomatis* and/or *M. genitalium* positivity when compared with BV-associated microbiota, while *L. iners* domination was not associated with presence/absence of these STIs.

This is the first study that provides strong evidence regarding associations between BV and *C. trachomatis*, *M. genitalium*, and *T. vaginalis* infections in women with very low risks for STIs, and it is also one of few studies investigating BV as a risk factor for *M. genitalium* infection. Although the examined women represented a subpopulation of outpatients attending the Ott Institute requesting or being offered testing for STIs, the frequency of STI detection was very low, ranging from 0.2% (*N. gonorrhoeae*) to 1.4% (*C. trachomatis*). Positivity for an STI is a strong epidemiological risk factor for concurrent and subsequent STI [12, 28, 41]. In a previous study [28], incident BV among STI clinic attendees preceded the acquisition of gonorrhea/chlamydia, and gonorrhea/chlamydia appeared to precede the acquisition of BV, which suggests that STIs and BV occur concurrently. In populations with a high risk for STIs, this creates a bias toward increased association between BV and STIs; therefore, we focused on a low-risk population. Our results show a strong association between BV-associated microbiota and infections with *C. trachomatis*, *M. genitalium*, and especially *T. vaginalis*.

Associations between BV and *C. trachomatis* and/or *T. vaginalis* have been investigated in many studies; however, only single studies have focused on BV and associations with *M. genitalium*, with conflicting evidence obtained [20, 22, 42, 43]. In some studies [22, 42], *M. genitalium* was less frequently detected in women with BV than in those without BV, whereas BV was an independent risk factor for the infection in other studies [43]. Most recently, prior BV was associated with a 3.5-fold increase in odds of incident *M. genitalium* in a cohort study [20], thereby suggesting that BV may enhance susceptibility to also *M. genitalium* infection. This finding was supported by our results where the odds of detecting *M. genitalium* was nearly 3 times higher in women with BV-associated microbiota than in women with normal microbiota.

The biological, including molecular, mechanisms by which vaginal microbiota affects the risk of STI acquisition remain largely unknown. It is believed that lactic acid and low pH are the main factors providing protection against STIs. A difference in the protective potential against STIs between different *Lactobacillus* species has been recently suggested, i.e., particularly *L. iners*-dominated vaginal microbiota has been associated with an increased *C. trachomatis* risk [16, 18, 31]. These results cannot be directly compared with our findings, because different molecular methods of defining microbiota types were used. Furthermore, since stratification of the cases by the individual infections would have resulted in less statistical power (due to the larger number of microbiota categories), we restricted this analysis to the combination of *C. trachomatis* and *M. genitalium* infections (single *T. vaginalis* infections were excluded because comprised exclusively BV-associated microbiota). The odds of STI detection in samples with *L. iners*-dominated microbiota was twice as high as that in *L. crispatus*-dominated microbiota, but twice as low as in those with BV-associated microbiota (non-significant). The dominance of the other three main *Lactobacillus* species was unambiguously negatively associated with STI presence. Most recently, it was demonstrated that lactic acid plays a major role in the anti-*C. trachomatis* properties of the vaginal microbiota by modulating host epithelial functions [44]. Moreover, D(−) lactic acid isomer provided higher protection than L(+) isomer, which is consistent with the decreased anti-*C. trachomatis* properties of *L. iners* that does not produce D(−) isomer [44]. Accordingly, enhanced understanding of the host response to the cervicovaginal microbiota is crucial, which will contribute to the development of strategies modulating natural protection against STIs.

In this study, analyzing repository samples, the only option for defining BV-associated vaginal microbiota was using a molecular test. The used test, AmpliSens Florocenosis/Bacterial vaginosis-FRT, has demonstrated a sensitivity of 93% and specificity of 86% when compared to the Nugent score and wet mount microscopy [33], and a sensitivity of 98–100% and specificity of 91% compared to the Amsel criteria and 454 16S rRNA gene pyrosequencing [32]. When compared with the Amsel criteria, Nugent score, culture and another PCR-based assay, BD MAX Vaginal Panel (BD Diagnostics), the AmpliSens Florocenosis/Bacterial vaginosis-FRT test had highest agreement with 16S rRNA gene sequencing [34]. Besides BV-associated vaginal microbiota and normal microbiota, the AmpliSens Florocenosis/Bacterial vaginosis-FRT test identifies “intermediate microbiota” (*G. vaginalis*/*A. vaginae* concentration plus *Lactobacillus* spp. concentration high) and “unspecified microbiota alteration” (*G. vaginalis*/*A. vaginae* concentration plus *Lactobacillus* spp. concentration low). Our data, based on a low number of samples, suggest that abnormal non-BV microbiota types do not significantly contribute to STI positivity.

The main limitations of our study are associated with the retrospective case-control design, with cross-sectional analysis of samples, which makes it impossible to determine causality. A longitudinal cohort study with sufficiently frequent testing for BV, enabling to detect BV prior to STI acquisition, would make it possible to establish causal relation between BV and STIs; however, it will be exceedingly challenging to achieve sufficient sample size to observe STI outcomes in a population with low STI positivity. A molecular test for the evaluation of the vaginal microbiota could enable to avoid prospective testing of all patients at every visit for BV-associated microbiota and to restrict the analysis to the samples obtained immediately prior to STI acquisition. Ideally, the molecular method should both differentiate and quantify the bacterial community state types and accurately define BV-associated microbiota. Furthermore, relevant patient data were lacking, e.g., regarding recent antimicrobial use. To limit this bias, we excluded women who had any urogenital samples positive for the four STIs and/or BV within 2 months prior to enrollment, and any antimicrobial use did likely not differ significantly in STI-positive versus STI-negative patients. We were additionally lacking information on sexual activity, STI history, and other potential confounders. However, controlling for age, one of the strongest confounders, did not significantly change the association between BV-associated microbiota and the STIs. Furthermore, we used samples submitted for STI testing rather than for vaginal microbiota evaluation. According to our clinical practice, the major specimen for *C. trachomatis* and *M. genitalium* detection in women is cervicovaginal sample. For *T. vaginalis* detection, vaginal swabs are mostly used. Nevertheless, it has been shown that the microbiota in the cervix and the vagina is generally similar [16, 45]. The cervical microbiota might be less abundant in bacteria than the vaginal microbiota, but we assessed relative rather than absolute bacterial loads, and we did not include samples with low bacterial load (< 6 lg geq/ml); therefore, it appears unlikely that the difference in the bacterial abundance between the cervix and vagina has largely affected the results. Finally, due to the few *N. gonorrhoeae* positive samples, no associations between BV and gonorrhea could be examined. In a recent meta-analysis, measures of association between BV and gonorrhea in the 8 included studies ranged from 0.80 to 3.75, with only one study showing a significant association [10]. Consequently, more data is imperative.

In conclusion, we show a significant, age-independent association between BV-associated microbiota and *C. trachomatis*, *M. genitalium*, and *T. vaginalis* infections also in women with low risk for STIs. Our findings suggest that the normal vaginal microbiota dominated by *L. crispatus, L. gasseri*, and *L. jensenii* is a strong protective factor against STIs, while *L. iners*-dominated microbiota is not significantly associated with STI outcomes. Our results confirm that STI prevention strategies should include interventions that reduce the incidence of BV and promote a protective vaginal microbiota.
